# The Role of Non-Invasive Brain Modulation in Identifying Disease Biomarkers for Diagnostic and Therapeutic Purposes in Parkinsonism

**DOI:** 10.3390/brainsci14070695

**Published:** 2024-07-12

**Authors:** Daniele Birreci, Martina De Riggi, Davide Costa, Luca Angelini, Antonio Cannavacciuolo, Massimiliano Passaretti, Giulia Paparella, Andrea Guerra, Matteo Bologna

**Affiliations:** 1 Department of Human Neurosciences, Sapienza University of Rome, Viale dell’Università, 30, 00185 Rome, Italy; daniele.birreci@uniroma1.it (D.B.); martina.deriggi@uniroma1.it (M.D.R.); massimiliano.passaretti@uniroma1.it (M.P.); giulia.paparella@uniroma1.it (G.P.); 2 IRCCS Neuromed, Via Atinense, 18, 86077 Pozzilli, IS, Italy; davide.costa@uniroma1.it (D.C.); luca.angelini@uniroma1.it (L.A.); antonio.cannavacciuolo@uniroma1.it (A.C.); 3 Department of Clinical Neuroscience, Karolinska Institutet, 17177 Stockholm, Sweden; 4 Parkinson and Movement Disorders Unit, Study Centre on Neurodegeneration (CESNE), Department of Neuroscience, University of Padua, 35121 Padua, Italy; andrea.guerra@unipd.it; 5 Padova Neuroscience Centre (PNC), University of Padua, 35121 Padua, Italy

**Keywords:** Parkinson’s disease, atypical Parkinsonism, non-invasive brain stimulation, transcranial magnetic stimulation, transcranial electrical stimulation, clinical neurophysiology

## Abstract

Over the past three decades, substantial advancements have occurred in non-invasive brain stimulation (NIBS). These developments encompass various non-invasive techniques aimed at modulating brain function. Among the most widely utilized methods today are transcranial magnetic stimulation (TMS) and transcranial electrical stimulation (TES), which include direct- or alternating-current transcranial stimulation (tDCS/tACS). In addition to these established techniques, newer modalities have emerged, broadening the scope of non-invasive neuromodulation approaches available for research and clinical applications in movement disorders, particularly for Parkinson’s disease (PD) and, to a lesser extent, atypical Parkinsonism (AP). All NIBS techniques offer the opportunity to explore a wide range of neurophysiological mechanisms and exert influence over distinct brain regions implicated in the pathophysiology of Parkinsonism. This paper’s first aim is to provide a brief overview of the historical background and underlying physiological principles of primary NIBS techniques, focusing on their translational relevance. It aims to shed light on the potential identification of biomarkers for diagnostic and therapeutic purposes, by summarising available experimental data on individuals with Parkinsonism. To date, despite promising findings indicating the potential utility of NIBS techniques in Parkinsonism, their integration into clinical routine for diagnostic or therapeutic protocols remains a subject of ongoing investigation and scientific debate. In this context, this paper addresses current unsolved issues and methodological challenges concerning the use of NIBS, focusing on the importance of future research endeavours for maximizing the efficacy and relevance of NIBS strategies for individuals with Parkinsonism.

## 1. Introduction

Parkinson’s disease (PD) and atypical Parkinsonism (AP) impose significant challenges for individuals and healthcare systems globally, manifesting in a wide-ranging spectrum of motor and non-motor symptoms that profoundly impact patients’ quality of life [1,2,3]. From bradykinesia and rigidity and/or tremor to cognitive impairment and autonomic dysfunction, the complexity of these conditions underscores the multifaceted nature of their impact on patients’ well-being [1,4,5]. Moreover, the chronic and progressive nature of PD and AP further exacerbates the burden, necessitating comprehensive approaches to diagnosis, management, and care [4,6,7]. In response to these complex challenges, non-invasive brain stimulation (NIBS) techniques have emerged as potential tools [8,9]. By modulating neural activity without invasive procedures, NIBS offers insights into the underlying pathophysiology and holds promise for developing targeted therapeutic interventions for individuals with Parkinsonism [10,11].

This paper aims to comprehensively discuss the current landscape of NIBS techniques in the context of PD and AP, emphasizing their role in identifying neurophysiological biomarkers for diagnostic and prognostic purposes [12,13]. Understanding the neural correlates of disease pathology and progression is essential for refining diagnostic criteria, predicting disease trajectories, and optimizing treatment strategies [8,10,14]. Furthermore, this paper seeks to summarize the therapeutic applications of NIBS, particularly assessing its effectiveness in alleviating motor symptoms in affected individuals [15,16,17,18]. However, the complex and heterogeneous nature of PD and AP underscores the importance of adopting a personalized and integrated approach to treatment tailored to each patient’s unique needs and encompassing both pharmacological and non-pharmacological interventions to optimize therapeutic outcomes [19,20].

This paper endeavours to significantly contribute to our understanding of NIBS techniques and their implications for managing PD and AP, through an exhaustive analysis of the latest research findings and clinical advancements. For this purpose, we searched for references in the PubMed databases without any date restriction. Database searches were limited to articles published in English. Key terms used to conduct the literature search were chosen and combined with the following English terms and their equivalents: “Non-invasive brain stimulation”, “biomarker”, and “Parkinson’s disease”. We reviewed the selected papers, screened the titles and abstracts, and focused on findings that indicated potential clinical utility as diagnostic biomarkers and possible therapeutic applications. We attempted to select primarily those studies that included a large sample of patients or followed a rigorous methodology, to avoid excessively confusing and uninformative results.

Finally, this paper aims to bridge the gap between scientific knowledge and practical application in neurodegenerative disorders by providing valuable insights that can inform future research directions and clinical practice. 

## 2. Overview of Non-Invasive Brain Stimulation

NIBS techniques encompass various methodologies designed to modulate cortical activity without surgical intervention, offering promising avenues for clinical and research applications [21,22]. The most widely utilized techniques are transcranial magnetic stimulation (TMS) and transcranial direct current stimulation (tDCS), each with unique mechanisms and applications [21,23,24,25].

TMS delivers short current pulses through a coil positioned on the scalp, generating a transient magnetic field in targeted brain regions and, in turn, producing an electrical current that causes neuronal depolarization [21,26,27]. Single-pulse TMS is a valuable tool for exploring cortical excitability and mapping cortical motor areas, thus providing insights into the brain’s functional organization [28,29]. Paired-pulse TMS allows the assessment of the excitability of various intracortical circuits, which are sustained by the activity of inhibitory and excitatory neurotransmitters, including GABA, acetylcholine, and glutamate [30,31]. Additionally, repetitive TMS (rTMS) enables long-lasting alterations in neural activity, offering opportunities for assessing brain plasticity mechanisms, including long-term potentiation (LTP), depression-like plasticity (LTD), and spike-time-dependent plasticity [32]. Also, by delivering repetitive pulses at specific frequencies or with patterned protocol designs, rTMS can induce plastic changes in cortical circuits [33]. Accordingly, research trials have applied repeated stimulation sessions with therapeutic purposes to test the potential of rTMS in ameliorating motor deficits and cognitive impairments associated with neurological disorders [14,18,34,35].

tDCS involves administering low-amplitude electrical currents through scalp electrodes, altering neuronal membrane potentials and consequently influencing cortical excitability patterns [23]. Anodal tDCS typically enhances cortical activity via depolarizing neurons, while cathodal tDCS exerts inhibitory effects through hyperpolarizing neurons [36]. Moreover, tDCS can induce longer-lasting effects than TMS, making it a promising tool for neurorehabilitation and cognitive enhancement [11,23].

Beyond TMS and tDCS, emerging NIBS techniques such as transcranial alternating current stimulation (tACS), transcranial random noise stimulation (tRNS), and transcranial ultrasound stimulation (TUS) offer novel approaches to modulating cortical activity [37]. tACS delivers an alternating current at specific frequencies, entraining neural oscillations and modulating network dynamics implicated in various cognitive processes [38,39]. In a different approach, tRNS involves the application of random noise currents to the scalp, enhancing cortical excitability and promoting synaptic plasticity [40,41,42]. Finally, TUS can noninvasively excite or inhibit neural activity in targeted deep brain regions by delivery of pulsed ultrasonic waves [43,44,45]. Given its excellent compatibility with non-invasive brain mapping and neuromodulatory techniques, systemic TUS effects can readily be assessed in basic and clinical research [44,46,47,48].

The diverse range of NIBS techniques provides researchers and clinicians with powerful tools to investigate brain function, explore the mechanisms underlying neurological disorders, and develop innovative therapeutic strategies. In this context, it is worth highlighting that numerous studies using NIBS have been carried out on patients with Parkinsonism [19].

Although the outcomes of these studies have provided variable results, they carry considerable significance from a translational perspective. This is because the abnormalities observed in human patients align with those previously demonstrated in experimental animal studies. For example, there were notable reductions in dendritic spine enlargement in Parkinsonian animals [49]. Conversely, animals with levodopa-induced dyskinesia showed increased dendritic spine enlargement [50]. These findings suggest that maladaptive changes in the plasticity of the primary motor cortex (M1) and disruptions in corticostriatal synaptic transmission play a central role in the pathophysiology of Parkinsonian disorders [51,52,53,54]. As our understanding of the brain continues to advance, NIBS holds great promise in shaping the future of neuroscience and clinical neurology, offering new insights into brain plasticity, cognition, and behaviour.

## 3. Neurophysiological Biomarkers in Parkinsonism

An essential aspect of research into NIBS involves assessing reliable neurophysiological biomarkers for diagnosing, staging, and monitoring disease progression and treatments in PD and AP.

### 3.1. Diagnostic Biomarkers

Paired-pulse TMS-derived measures like short-interval intracortical inhibition (SICI) and short-latency afferent inhibition (SAI) are particularly interesting as diagnostic biomarkers; they may prove helpful in differentiating between patients with various forms of Parkinsonism [12,21,51]. In particular, assessments of intracortical excitability—such as SICI and SAI—offer insights into underlying GABAergic and cholinergic neurotransmission changes, respectively [21,55].

A notable aspect worth highlighting is that, in some instances, neurophysiological measurements can provide compelling evidence that reflects underlying anatomopathological changes. The relationship between neurophysiological measures and the underlying anatomopathological changes is particularly evident in the case of SICI because it can mirror the loss of inhibitory interneurons, a hallmark often associated with tau pathology in various brain regions [56]. This relationship has been demonstrated in animal models and human studies, particularly in patients diagnosed with progressive supranuclear palsy (PSP) [56,57]. In these cases, SICI measures appear to correlate with the extent of neurodegeneration and the presence of tau-related lesions [58,59]. Interestingly, SICI is impaired in PD from the earliest stage of the disease, even in the asymptomatic side of highly asymmetric de novo patients [60,61]. This result strengthens the idea that SICI abnormality may reflect anatomopathological changes occurring even before clinically overt Parkinsonism. However, another hypothesis is that cortical disinhibition (as indicated by impaired SICI) represents a compensatory mechanism of cortical motor areas to counteract defective basal ganglia output and motor symptoms in PD [60,62,63,64]. This idea is also supported by recent evidence from NIBS experiments showing that SICI reduction dynamically occurs in patients who manifest bradykinesia amelioration during tACS [64,65]. These findings suggest that certain neurophysiological measurements could serve as valuable indicators of specific pathological processes, opening a pathway for more targeted diagnostic and therapeutic approaches. However, the clinical applications of neurophysiological biomarkers remain limited by the lack of methodological standardization and established cut-offs, thus preventing integration with validated biomarkers for diagnostic and prognostic purposes [66,67]. As research continues to evolve, SICI and similar metrics may offer a window into the complex mechanisms of neurodegenerative diseases, providing a bridge between clinical assessment and underlying brain pathology.

Another important point to consider is that, despite the variability often observed in TMS measurements, a promising approach involves analyzing multiple intracortical circuits in the same patient. This method may prove useful in differentiating between various forms of AP. In one study, the authors identified significant alterations in SICI among patients with different Parkinsonian disorders: PSP, corticobasal syndrome (CBS), and dementia with Lewy bodies (DLB) [57]. They also found substantial changes in SAI among patients with Alzheimer’s disease (AD) and DLB [57]. They constructed a decision tree analysis using these specific alterations, leading to relatively high diagnostic accuracy, especially for CBS and PSP [57]. Overall, the study demonstrated that TMS measurements could play a critical role in the differential diagnosis of AP. Even though there is still no available data about the performance of such methods in discriminating PD from AP, this approach is interesting and promising. Indeed, by targeting specific neurophysiological abnormalities, clinicians may be able to distinguish between various neurodegenerative disorders with greater accuracy, thereby improving diagnosis and treatment outcomes for patients with these complex conditions. In this regard, short-interval intracortical facilitation (SICF), a paired-pulse TMS measure that mainly reflects glutamatergic activity and is altered in PD [68,69,70], is normal in PSP patients [71]. Future studies may allow the upgrade of the current algorithm by adding data on the activity of multiple intracortical circuits in PD and other AP.

rTMS and tDCS protocols allow the induction and assessment of brain plasticity mechanisms [32]. Converging evidence from studies that adopted various NIBS protocols suggests that LTP-like plasticity is impaired in PD, i.e., brain excitability does not increase after applying the protocol [72,73,74]. However, there is considerable variability in NIBS after-effects, possibly due to inter-individual variability, different disease characteristics, and methodological issues. Moreover, although only limited evidence exists about possible abnormal plasticity in AP, many studies have highlighted generally impaired LTP-like plasticity processes, as in multiple system atrophy (MSA), CBS, and PSP [12,75]. Finally, defective plasticity mechanisms are also known to be present in numerous other neurological conditions [76,77,78,79]. All these factors prevent using brain plasticity-altered measures as a reliable disease biomarker in PD or AP. An overview of the main neurophysiological diagnostic biomarkers in Parkinsonism is summarized in Table 1.

### 3.2. Staging and Progression Biomarkers

NIBS techniques offer valuable insights into both the staging and progression of Parkinsonian disorders. A large cross-sectional study on PD showed that SICI remained altered regardless of disease stage, thus questioning its potential utility as a biomarker of disease progression [60]. Similarly, the loss of LTP-like plasticity is a neurophysiological feature present from the early stages of PD and does not worsen as the disease advances [72,73,74]. However, SICF emerges as a more stage-dependent neurophysiological abnormality. SICF is abnormally enhanced in the early stages of PD, and it becomes increasingly altered as the disease progresses [68].

This alteration is particularly pronounced in patients who develop levodopa-induced dyskinesia (LID), a common complication in advanced PD [80]. Studies have shown that SICF levels are higher in patients with LID than those without and that these changes correlate with the severity of LID [69]. Also, modifications in LID severity over time are related to changes in the excitability of SICF-dependent circuits. This supports the evidence observed in animal models, where changes in glutamatergic pathways were connected to similar patterns of dyskinesia [50,81,82]. Moreover, these data suggest a link between SICF alterations and the progression of motor symptoms and complications [83]. Another possible neurophysiological biomarker of LID is the loss of bidirectional plasticity [84,85]. This phenomenon can be tested in humans by measuring the ability of the M1 to return to baseline excitability levels after applying an LTP-like plasticity protocol (‘depotentiation’). In PD, the TBS-induced LTP-like effects can be depotentiated by a specific rTMS protocol only in patients without LID, while patients with LID are ‘resistant’ to depotentiation [52]. Further emphasizing the possible specificity of this abnormality for LID pathophysiology, subtle depotentiation deficits have even been found to predict LID onset in PD patients [86].

Recent studies have also used kinematic analysis techniques to explore the relationship between neurophysiological changes and specific movement alterations [51,61,87]. These studies suggest that neurophysiological parameters can act as indirect biomarkers for the severity of bradykinesia in PD and other neurodegenerative diseases, providing valuable information on disease progression [51,77,87]. However, the reliance on indirect evidence limits this approach. Longitudinal studies offer a complementary perspective, allowing researchers to track changes over time. One study demonstrated that plasticity alterations in the M1 evolved in parallel with the worsening of motor symptoms in PD, indicating a dynamic progression of the underlying pathophysiology [53]. The challenge with longitudinal studies, however, lies in their complexity and the risk of participant dropout, which can affect the continuity and reliability of the data. These collective findings underscore the potential of TMS measures as tools for understanding and monitoring the progression of Parkinsonian disorders [53]. By combining cross-sectional and longitudinal approaches, researchers can gain a more comprehensive view of the neurophysiological changes associated with these diseases, ultimately leading to better diagnostic and therapeutic strategies.

### 3.3. Estimating and Monitoring the Effects of Anti-Parkinsonian Drugs

Several studies have used single- and paired-pulse TMS to assess the cortical effects of anti-Parkinsonian drugs. Despite the cardinal pharmacological treatment for PD being levodopa, its effects on neurophysiological M1 abnormalities are variable [51,88]. Converging evidence suggests that the abnormally increased overall corticospinal excitability, as estimated according to the input–output (I-O) curve steepness using single-pulse TMS, is reduced by levodopa administration in PD [51,69,88]. This parameter might thus be applied to verify the responsiveness to levodopa in patients with Parkinsonism. However, to date, no study has specifically tested whether cortical responsiveness to levodopa may predict the drug’s clinical effectiveness in individual PD and AP cases. We believe this could be an interesting topic to address in future studies.

In contrast to the overall corticospinal excitability, defective GABA-A-ergic, and enhanced glutamatergic intracortical activity, as assessed via SICI and SICF, respectively, are scarcely responsive to oral levodopa intake in PD [51,53,68,69,70,89,90]. This aspect limits the possible use of SICI and SICF as NIBS biomarkers to monitor the effect of levodopa intake in patients. Interestingly, a recent study showed that, unlike oral intake, levodopa–carbidopa intestinal gel (LCIG) infusion therapy, which ensures continuous dopaminergic stimulation [91], significantly improves the altered SICI in advanced PD patients. Moreover, changes in the levels of SICI are correlated with clinical improvements in dyskinesia and motor fluctuations [54]. SICI could thus be used as a biomarker to estimate and monitor the clinical effects of LCIG over time.

In recent years, SICF has been proven a potentially useful NIBS measure to assess, monitor, and possibly predict the clinical–neurophysiological effects of safinamide, a monoamine oxidase-type B (MAO-B) inhibitor that also blocks voltage-gated sodium channels and glutamate release when used at high dosage [69,83,92,93]. Indeed, in PD patients with LID, safinamide reduced the abnormally enhanced SICF in a dose-dependent manner, normalizing this alteration at a high dosage [69]. This effect, reflecting a downregulation of overactive intracortical glutamatergic activity, was already present after 2 weeks of treatment and persisted after 1 year of chronic therapy [69,83]. Furthermore, the level of SICF reduction was related to the beneficial effects of safinamide on dyskinesia severity over time [83]. These data led to the hypothesis that SICF could be a biomarker of neurophysiological and clinical response to safinamide in PD patients.

## 4. Therapeutic Applications of Non-Invasive Brain Stimulation

Beyond their utility in biomarker discovery, NIBS techniques offer promising therapeutic avenues for individuals with Parkinsonism. Among these techniques, rTMS has garnered considerable attention as a potential treatment modality for ameliorating motor symptoms such as tremors and bradykinesia, which are hallmark features of PD and AP [10,17,19,94]. However, the outcomes of studies investigating the efficacy of rTMS in motor symptom management have been heterogeneous, with varying degrees of success reported across different trials. While some clinical trials reported significant improvements in motor function following rTMS interventions, others yielded inconclusive findings or demonstrated only modest benefits [18,95]. These discrepancies may have stemmed from differences in study design, patient populations, and stimulation protocols [96,97]. Additionally, the underlying pathophysiological mechanisms of PD and AP are complex and multifaceted, further complicating the interpretation of treatment outcomes [73]. Overall, to date, the most effective therapeutic rTMS paradigm for ameliorating cardinal motor symptoms in PD is high-frequency, i.e., excitatory, rTMS on the M1, which, however, is not FDA-approved [10,18]. Regarding rTMS in AP, neurophysiological studies involving multiple stimulation sessions are difficult to conduct since these diseases are rare and more severe than PD. Accordingly, large randomized controlled trials are still lacking in the literature [95]. Traditional transcranial electrical stimulation techniques, i.e., tDCS, have been repeatedly applied over motor cortical areas to improve motor disturbances in PD. Anodal tDCS on the M1 is the protocol that has generally produced better results, but the conclusions of different studies have generally been highly variable [98]. In line with this idea, a meta-analysis that only considered the outcomes of randomized controlled trials showed no superiority of real tDCS over sham stimulation in terms of Movement Disorders Society-sponsored version of the Unified Parkinson Disease Rating Scale (MDS-UPDRS) part III scores [99]. tDCS application in AP suggested possible beneficial effects on specific motor symptoms, like gait in MSA and language disturbances in PSP patients [100,101]; however, the very low number of studies limits these promising results [17,24]. A comparison between rTMS and tDCS was recently performed in a meta-analysis investigating the effect of these two techniques on walking and balance ability in PD, targeting mostly the M1 and the dorsolateral prefrontal cortex (DLPFC). Both techniques demonstrated an improvement in MDS-UPDRS-III scores and variables associated with the ability to walk, such as step width, cadence, six-minute walking test (6 MWT), and the timed up-and-go test (TUGT), with a more significant result with rTMS rather than tDCS [102].

Recent studies have explored innovative applications of NIBS in ameliorating the key motor symptoms of PD by targeting the M1. In two experiments, researchers investigated the therapeutic potential of tACS in this area using different stimulation frequencies. They found that beta-frequency stimulation led to a decline in motor performance, while gamma-frequency stimulation had the opposite effect, indicating potential motor improvement [64,65]. The positive effects of gamma-tACS were related to the modulation of SICI. This aligns with the idea that GABAergic mechanisms play a pivotal role in this context [65,103]. These studies suggest that SICI could be a biomarker to predict patients’ responsiveness to tACS, offering a personalized approach to treatment [60,104]. tACS has also been applied to the M1 to suppress rest tremor in PD. Indeed, from a pathophysiological point of view, tremor is thought to be generated by a central pathologically oscillating network where the M1 is a crucial node [105]. In a seminal study, M1-tACS was applied at the tremor frequency and at a specific phase lag from the ongoing tremor, individualized for each patient. This approach resulted in a 21–53% reduction in tremor amplitude across patients [106]. Unfortunately, other groups have not replicated these data so far.

In addition to the M1, other areas are being explored as therapeutic targets in PD and AP. For example, continuous theta burst stimulation (cTBS), a specific form of patterned rTMS delivered over the cerebellum, has been tested for its effects on rest tremor in PD, with mixed results [107]. Although initial trials found no significant reduction in tremor severity, more recent neuroimaging studies suggest that the cerebello–thalamo–cortical circuit may be crucial in generating rest tremors in PD [108,109]. This raises the possibility that cerebellar NIBS could be effective as an add-on therapy in some patients [110]. Cerebellar cTBS has also been proven effective in reducing LID, another challenge in PD treatment that has only limited pharmacological approaches [111]. In a previous study, a 2-week course of cerebellar cTBS induced persistent clinical beneficial effects for up to 4 weeks after the end of the stimulation period [112]. Other possible applications of cerebellar TBS in PD are currently being defined, exploiting the cerebellum’s role in several clinical features of the disease [113,114,115]. Only a few studies have investigated the effect of cerebellar rTMS in patients with AP, focusing particularly on PSP, in which cerebellar stimulation improved patients’ stability and speech [116,117].

Another promising NIBS target in PD is the pre-supplementary motor area (pre-SMA). Research shows that levodopa can cause overactivation of the pre-SMA in patients with peak-of-dose dyskinesia, and low-frequency, i.e., inhibitory, rTMS aimed at this region was demonstrated to be effective in reducing dyskinesia severity in a recent study [16]. Interestingly, the modulation of pre-SMA activity induced by rTMS, as measured using functional magnetic resonance imaging (fMRI), was linearly related to improving dyskinesia severity [16,52,118,119]. This again underscores the potential role of neuroimaging biomarkers in guiding NIBS therapy.

In summary, despite their potential, the interpretation of each technique/target area combination remains quite enigmatic, presenting promising results in some studies and inconclusive results in others. The combinations of the main NIBS techniques and principal targets are summarized in Table 2. This inconsistency’s prime sources are various methodological challenges, including heterogeneous TMS settings, variability in stimulation parameters, and small sample sizes in many studies [96]. A major obstacle in the clinical application of rTMS for PD and AP is the lack of a clear rationale for choosing specific cortical stimulation sites [13,120]. Additional complicating factors include technical variations in stimulation frequency and intensity, train durations, inter-train intervals, and session counts [21,27,96]. Moreover, potential drug interactions and inconsistent TMS dosages can affect outcomes [30]. Another critical aspect is our limited understanding of the underlying mechanisms by which rTMS affects humans.

Despite the advances, the influence of existing treatments on NIBS outcomes requires careful consideration. NIBS is typically used as an add-on therapy, and the interaction between orally administered medications and TMS measures can lead to non-linear effects [11]. For example, tDCS has shown promise as an adjunctive therapeutic intervention for individuals with PD and AP. tDCS can modulate cortical excitability and potentially alleviate motor symptoms by delivering low-amplitude electrical currents to targeted brain regions [122,123,124]. However, when combined with conventional pharmacotherapy or physical or cognitive training, tDCS may offer synergistic effects, enhancing the efficacy of standard treatment regimens [125,126]. A paradigmatic example in this regard comes from a recent study where bilateral M1 tDCS was combined with a 4-week rehabilitation program for treating Pisa syndrome in PD, and this combined approach led to much greater improvements in postural alterations compared with rehabilitation alone [127]. Similarly, a double-blind, randomized, sham-controlled study demonstrated that high-frequency rTMS delivered over the M1 leg area associated with treadmill training could boost the training effects, improving gait parameters for up to 3 months post-intervention [128].

Further complicating matters, current data on neurophysiological effects in advanced PD stages are limited, necessitating more research on how deep brain stimulation (DBS) and other treatments might alter cortical excitability and plasticity [129,130]. These findings highlight the challenges and potential pathways for using NIBS in treating PD and related movement disorders. As research continues to evolve, a clearer understanding of the mechanisms, optimal stimulation parameters, and interaction with other treatments will be critical in realizing the full therapeutic potential of NIBS in clinical practice.

The optimization of stimulation parameters and the delineation of optimal target regions for NIBS interventions remain areas of active investigation and debate within the scientific community. Factors such as the intensity, duration, and frequency of stimulation and the precise localization of target brain areas can significantly influence treatment outcomes. Moreover, individual variability in treatment response and disease progression further complicates the development of standardized protocols for NIBS-based therapies.

Despite these challenges, ongoing research efforts continue to refine our understanding of the therapeutic potential of NIBS techniques in PD and AP. By elucidating the underlying mechanisms of action and optimizing treatment protocols, researchers aim to maximize the clinical benefits of NIBS interventions for individuals affected by these debilitating neurological disorders. Through collaborative research endeavours and rigorous clinical trials, the field of NIBS holds promise for delivering innovative and effective treatments that improve the quality of life for patients with PD and AP.

## 5. Challenges and Future Directions

Despite the burgeoning interest in NIBS techniques for PD and AP, several challenges limit their widespread adoption and clinical efficacy. These obstacles represent critical areas for improvement to maximize the potential of NIBS as a therapeutic modality for individuals with these debilitating neurological disorders.

One significant challenge is the interindividual variability in treatment response to NIBS interventions. While some patients may exhibit substantial improvements in motor symptoms or cognitive function following NIBS treatment, others may show minimal or negligible responses [96]. This variability can be attributed to many factors, including differences in disease severity, underlying neuropathology, and individual neuroanatomical variability. Moreover, demographic factors such as age, sex, and genetic predisposition may further influence treatment outcomes [21,131]. Addressing this variability requires a comprehensive understanding of the factors contributing to individual differences in treatment response and the development of personalized treatment strategies tailored to each patient’s unique profile.

Another challenge is the lack of standardized protocols for NIBS interventions in PD and AP. Variation in stimulation parameters, such as intensity, duration, and frequency, across different studies, makes it challenging to compare results and draw definitive conclusions about treatment efficacy. Establishing consensus guidelines for NIBS protocols, informed by rigorous preclinical and clinical research, is essential for ensuring the reproducibility and reliability of findings across studies. Standardized protocols will also facilitate the replication of successful interventions and the identification of factors contributing to treatment response variability.

Furthermore, there remains an incomplete understanding of the underlying neurobiological mechanisms mediating the effects of NIBS in PD and AP. While NIBS can induce changes in cortical excitability and neural network connectivity, the specific mechanisms by which these changes translate into clinical improvements remain poorly understood. Future perspectives to address this issue should include the execution of new studies adopting a multimodal assessment of patients, i.e., not only limited to NIBS measures but also recording behavioural data (e.g., kinematic analysis of movement or quantitative indexes of cognitive performance), neuroimaging, and biological markers. Future research endeavours should also prioritize elucidating the broader neurobiological effects of NIBS, including its possible impact on neurotransmitter systems, synaptic plasticity, neuroinflammatory processes, misfolded protein aggregation, and neurodegeneration [33,132]. Interestingly, preliminary research suggests an intricate landscape where NIBS can influence various neurobiological processes, including glial cell activity [14,133,134]. In this regard, one recent study in experimental animal models of Parkinsonism demonstrated how TBS can modify astrocyte function [135]. This finding suggests that the benefits of NIBS may extend beyond neuronal circuits, affecting other cellular components that play crucial roles in maintaining brain health and function. Researchers can develop more targeted and effective NIBS interventions for PD and AP by understanding these mechanisms more deeply.

Moreover, developing personalized treatment approaches grounded in neurophysiological biomarkers holds promise for optimizing therapeutic outcomes in PD and AP populations. In the last decade, significant advances have been made in understanding the pathophysiology of many signs and symptoms of PD and AP [105,136,137]. Therefore, future perspectives should include applying pathophysiologically driven NIBS approaches. For instance, modulating cortical oscillations instead of brain excitability should be advised when the targeted symptom is known to relate to altered oscillations rather than hypo/overactive brain regions. In addition, biomarkers, such as neuroimaging measures, electrophysiological parameters, and genetic and biological markers, can provide valuable insights into individual disease trajectories and treatment responses [138,139,140]. By incorporating these biomarkers into treatment algorithms, clinicians can tailor NIBS interventions to each patient’s needs, maximizing therapeutic efficacy and minimizing adverse effects. Possible future approaches to apply in this context include modulating the target choice based on the prevailing pathological node of the altered network (similar to what has been recently tested in Alzheimer’s disease [141]) or choosing the NIBS modality based on the individual patient profile.

## 6. Conclusions

**Figure 1:**
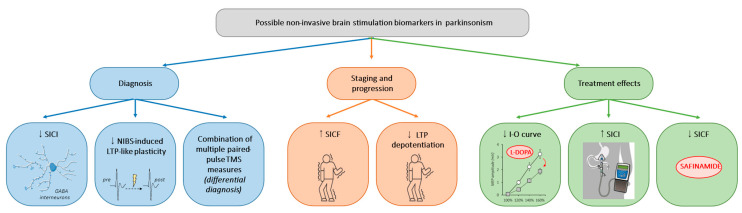
Possible non-invasive brain stimulation biomarkers in Parkinsonism. Illustration of the possible neurophysiological biomarkers for diagnosis, staging, and progression, and therapeutic applications in Parkinsonism. SICI: short-interval intracortical inhibition, NIBS: non-invasive brain stimulation, TMS: transcranial magnetic stimulation, SICF: short-interval intracortical facilitation, LTP: long-term potentiation, I-O curve: input–output curve.

One of the primary strengths of NIBS lies in its ability to offer insights into the intricate pathophysiological mechanisms underlying PD and AP. NIBS techniques allow researchers to probe neural circuits and elucidate various abnormalities associated with these disorders by modulating cortical activity non-invasively. Through neuroimaging, electrophysiological recordings, and other advanced techniques, NIBS studies have provided valuable translational evidence, showing a range of M1 abnormalities, including (i) decreased GABAergic and increased glutamatergic neurotransmission and (ii) maladaptive or disrupted bidirectional plasticity. Such findings offer the potential for developing new disease biomarkers that could aid in diagnosis, staging, and monitoring the response to therapeutic interventions. A summary of possible neurophysiological biomarkers in Parkinsonism is illustrated in Figure 1.

Additional studies are needed to confirm the reliability of these biomarkers across different stages of disease progression and in diverse patient populations. Furthermore, efforts to standardize experimental protocols and measurement techniques could help address concerns regarding the reproducibility of these biomarkers across different research settings. Again, NIBS holds great promise as a therapeutic modality for PD and AP, although several challenges must be addressed to realize its full potential. For example, variability in treatment response among individuals, methodological inconsistencies across studies, and gaps in our understanding of the long-term effects of NIBS interventions represent critical areas for further investigation. By addressing interindividual variability in treatment response, standardizing protocols, advancing our understanding of neurobiological mechanisms, and incorporating personalized treatment approaches, we can enhance the clinical utility of NIBS and improve outcomes for individuals living with PD and AP.

## Figures and Tables

**Table 1:** Neurophysiological diagnostic biomarkers in Parkinsonism. TMS: transcranial magnetic stimulation, rTMS: repetitive TMS, SICI: short-interval intracortical inhibition, SAI: short-latency afferent inhibition, SICF: short-interval intracortical facilitation, PD: Parkinson’s disease, DLB: dementia with Lewy bodies, PSP: progressive supranuclear palsy, CBS: corticobasal syndrome, MSA: multiple system atrophy.

NIBS Methods	Results	Major References
Paired-pulse—SICI	Reduced in PD, DLB, PSP, CBS.	[12,21,51,56,57,59,60,61]
Paired-pulse—SAI	Normal or reduced in PD.	[21]
	Reduced in DLB.	[57]
Paired-pulse—SICF	Increased in PD.	[68,69,70]
	Normal in PSP.	[71]
rTMS protocols—plasticity measures	Impaired LTP-like plasticity processes in PD, MSA, CBS, and PSP.	[12,51,73,75]

**Table 2:** Therapeutic applications of non-invasive brain stimulation (NIBS) techniques and possible stimulation targets in Parkinsonism. rTMS: repetitive transcranial magnetic stimulation, tDCS: transcranial direct current stimulation, tACS: transcranial alternating current stimulation, cTBS: continuous theta burst stimulation, M1: primary motor cortex, pre-SMA: pre-supplementary motor area, DLPFC: dorsolateral prefrontal cortex, PD: Parkinson’s disease, AP: atypical Parkinsonism, PSP: progressive supranuclear palsy, MSA: multiple system atrophy, LID: levodopa-induced dyskinesia.

NIBS Methods/Target Area	Results	Major References
rTMS/M1	Excitatory rTMS improved motor symptoms, i.e., bradykinesia and rest tremor, in PD patients.	[10,18,19,94,121]
	Not conclusive in AP patients.	[17,95]
rTMS/pre-SMA	Inhibitory rTMS improved dyskinesia severity in PD patients with peak-of-dose dyskinesia.	[16,52,118,119]
rTMS/Cerebellum	Cerebellar continuous theta burst stimulation (cTBS), a specific form of patterned rTMS, reduced rest tremor and LID in PD patients.	[107,111,112]
	Cerebellar rTMS improved patients’ stability and speech in PSP.	[116,117]
tDCS/M1	Mild improvement in walking and balance ability in PD patients.	[99,102]
	tDCS improved gait in MSA and language disturbances in PSP patients.	[100,101]
tDCS/DLPFC	Mild improvement in walking and balance ability in PD patients.	[99,102]
tACS/M1	tACS gamma-frequency stimulation of the M1 improved bradykinesia in PD patients.	[64,65]
	tACS at the tremor frequency and a specific phase lag from the ongoing tremor reduced tremor amplitude in PD patients.	[106]

## Abbreviations

Alzheimer’s disease (AD), atypical Parkinsonism (AP), continuous theta burst stimulation (cTBS), corticobasal syndrome (CBS), deep brain stimulation (DBS), dementia with Lewy bodies (DLB), dorsolateral prefrontal cortex (DLPFC), depression-like plasticity (LTD), functional magnetic resonance imaging (fMRI), input–output curve (I-O curve), levodopa-induced dyskinesia (LID), levodopa-carbidopa intestinal gel (LCIG), long-term potentiation (LTP), monoamine oxidase-type B (MAO-B), multiple system atrophy (MSA), Movement Disorders Society-sponsored version of the Unified Parkinson Disease Rating Scale (MDS-UPDRS), non-invasive brain stimulation (NIBS), Parkinson’s disease (PD), pre-supplementary motor area (pre-SMA), primary motor cortex (M1), progressive supranuclear palsy (PSP), repetitive transcranial magnetic stimulation (rTMS), short-interval intracortical facilitation (SICF), short-interval intracortical inhibition (SICI), short latency afferent inhibition (SAI), six-minute walking test (6 MWT), timed up-and-go test (TUGT), transcranial alternating current stimulation (tACS), transcranial direct current stimulation (tDCS), transcranial electrical stimulation (TES), transcranial magnetic stimulation (TMS), transcranial random noise stimulation (tRNS), transcranial ultrasound stimulation (TUS).
